# Dietary Inhibitors of Histone Deacetylases in Intestinal Immunity and Homeostasis

**DOI:** 10.3389/fimmu.2013.00226

**Published:** 2013-08-01

**Authors:** R. Schilderink, C. Verseijden, W. J. de Jonge

**Affiliations:** ^1^Tytgat Institute for Liver and Intestinal Research, Department of Gastroenterology and Hepatology, Academic Medical Center, Amsterdam, Netherlands

**Keywords:** HDAC, intestinal epithelium, inflammatory bowel diseases, short chain fatty acids, butyrate

## Abstract

Intestinal epithelial cells (IECs) are integral players in homeostasis of immunity and host defense in the gut and are under influence of the intestinal microbiome. Microbial metabolites and dietary components, including short chain fatty acids (acetate, propionate, and butyrate, SCFAs), have an impact on the physiology of IECs at multiple levels, including the inhibition of deacetylases affecting chromatin remodeling and global changes in transcriptional activity. The number and diversity of butyrate-producing bacteria is subject to factors related to age, disease, and to diet. At physiological levels, SCFAs are inhibitors of histone deacetylases (HDACs) which may explain the transcriptional effects of SCFAs on epithelial cells, although many effects of SCFAs on colonic mucosa can be ascribed to mechanisms beyond HDAC inhibition. Interference with this type of post-translational modification has great potential in cancer and different inflammatory diseases, because HDAC inhibition has anti-proliferative and anti-inflammatory effects *in vitro*, and in *in vivo* models of intestinal inflammation. Hence, the influence of dietary modulators on HDAC activity in epithelia is likely to be an important determinant of its responses to inflammatory and microbial challenges.

## The Intestinal Epithelium Contributes to Intestinal Homeostasis

The intestinal epithelial cells (IECs) act as a physical barrier between the stromal cells and immune cells in the lamina propria and luminal antigens. IECs contribute to intestinal homeostasis via secretion of cytokines, metabolites, and anti-microbial peptides. The intestinal epithelial layer in fact comprises specialized epithelia of many different cell types: Paneth cells that secrete different anti-microbial peptides, mucin secreting goblet cells, enteroendocrine cells, enterocytes, and colonocytes outside the crypt and M-cells, located on top of intestinal lymphoid follicles. Interspersed with the IECs are different immune cells, including intra-epithelial γδ-T cells and specialized mucosal macrophages with the capacity to sampling antigen from the lumen.

Intestinal epithelial cells express different pattern recognition receptors, including Toll-like receptors and NOD-like receptors but the tolerogenic phenotype of epithelia is not readily understood. For example, IECs acquire endotoxin tolerance toward lipopolysaccharide despite the expression of TLR4 soon after birth (1), in some aspects comparable to the TLR hyporesponsiveness found in intestinal macrophages (2). In support of intestinal homeostasis, IECs secrete many factors that are needed for homeostasis of the mucosal compartment including transforming growth factor beta (TGF-beta), IL-10, thymic stromal lymphopoietin (TSLP), retinoic acid (RA), prostaglandin E2 (PGE2). Some of these factors have prominent autocrine effects. TGF-beta, for example, suppresses proliferation of rat IECs thereby preventing hyperproliferation and tumor formation (3). Cao et al. showed that diet supplemented with 20% pectin (dietary fiber) could increase TGF-beta/SMAD3 signaling in mouse jejunum, but not in colon (4). TGF-beta is also a regulator of immune cell polarization and function, but these effects have been discussed elsewhere (5–7).

## Microbiome, Metabolites, and Epithelial Biology

Intestinal microbes have a mutualistic relationship with its host in part by dictating epithelial cell responses and intestinal barrier homeostasis. We coexist with our microbiota, but this relationship sometimes becomes pathological, and contributes to diseases such as inflammatory bowel disease (IBD). The impact of the gut microbiome on immune homeostasis has been identified around 60 years ago, but recent metagenomic analyses of microbiome changes associated with multiple diseases have advanced this field enormously (8–10). These recent screens have advanced our understanding of the changed microbiome in IBD (11, 12). A main outcome of microbiome sequencing is an underrepresentation of SCFA producing strains in IBD patients (13), corresponding with earlier observations of for instance decreased SCFA levels in feces of children with IBD (14).

**Figure 1:**
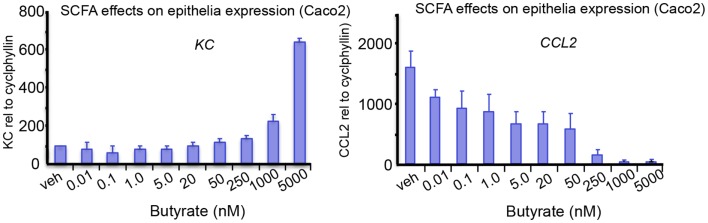
**The effects of SCFA butyrate on epithelial cell responses in Caco-2 enterocyte-like cells**. Cells were stimulated with 10 ng/mL of ILlb and tested against indicated butyrate concentrations added simultaneously. Expression levels of IL-8, and CCL2, were measured after 24 h treatment. Butyrate modulates the epithelial responses to cytokines at relevant concentrations. Tytgat Institute, 2013.

Carbohydrates, resistant to breakdown in the stomach and small intestine, are subject to colonic fermentation to result in the production of SCFAs, fatty acids containing 1–6 carbon atoms. Anaerobic bacteria generate the major SCFAs acetate, propionate, and butyrate and the highest production is found in the proximal colon. SCFA concentrations are dependent on the availability and source of substrate, the microflora, and gut transit time. The estimated total amount of SCFAs in the proximal colon is higher than in the distal colon (from 70–140 mM in proximal to 20–70 mM distal part), mainly because of availability of carbohydrates and presence of water (15, 16). Oxidation of SCFAs accounts for 60–70% of the colonic epithelial energy need (16). Butyrate is through β-oxidation the main, and preferred, energy source for colonocytes and also has a variety of other physiological effects, including modulation of epithelial responses to cytokines, see for instance Figure 1 and a summary of previous work on this matter in Table 1.

**Table 1:** **Examples of SCFA-induced effects on intestinal epithelium**.

SCFA	HDAC inhibition	Effects on intestinal epithelium		Tested in	Reference
Acetate	Inactive	HDAC inhibition		Nuclear extracts of HT-29	Waldecker et al. (17)
				HT-29	Kiefer et al. (18)
Propionate	Active, unknown	HDAC inhibition		Nuclear extracts of HT-29 Caco-2	Waldecker et al. (17), Sanderson (19)
		Transcription factor	Activation of AP-1 signaling pathway	HT-29 and caco-2	Nepelska et al. (20)
		Cell cycle	Reduced cell growth and inhibition of differentiation by decreased p21 and CB1 mRNA	HT-29, HCT-116	Hinnebusch et al. (21)
Butyrate	Class I and class IIa	HDAC inhibition		Nuclear extracts of HT-29	Waldecker et al. (17)
		Energy supply	Energy supply	Isolated human colonocytes	Roediger (22)
		Cell cycle	Reduced cell growth and inhibition of differentiation by decreased p21 and CB1 mRNA	HT-29, HCT-116	Hinnebusch et al. (21)
			Inhibition of proliferation	HT-29 cells	Siavoshian et al. (23)
			Stimulation of alkaline phosphatase and dipeptidyl peptidase IV activity		
		Transcription factor	Activation of AP-1 signaling pathway	HT-29 and caco-2	Nepelska et al. (20)
			Transactivation of Krüppel-like factor 4	HT-29	Chen et al. (24)
		Barrier function	Reduction of paracellular permeability	Caco-2	Mariadason et al. (25)
			Enhancement of barrier function by tight junction assembly facilitation	Caco-2	Peng et al. (26)
		Cytokine expression	Induction of A20 (negative regulation of NFkB), downregulation of IL-8	Fetal human intestinal epithelial cells and fetal intestinal explants	Weng et al. (27)
			Increased expression of IL-32α	HT-29, SW480, T84	Kobori et al. (28)
		Other	Enhanced expression of di/tripeptide transporter hepT1	Caco-2-BBE cells and *in vivo*	Dalmasso et al. (29)
			Up-regulated transcription of several mucin genes	Human goblet cell line HT-29-Cl.16E	Gaudier et al. (30)
Valerate	Active, unknown	HDAC inhibition		Caco-2	Sanderson (19)
		Cell cycle	Reduced cell growth and inhibition of differentiation by decreased p21 and CB1 mRNA	HT-29, HCT-116	Hinnebusch et al. (21)
Caproate	Active, unknown	HDAC inhibition		Caco-2	Sanderson (19)
	Inactive			HT-29, HCT-116	Hinnebusch et al. (21)

## Regulation of Epithelial Gene Expression through Acetylation and Deacetylation

In the large bowel, SCFAs butyrate and propionate reach concentrations that are able to inhibit the activity of an important class of epigenetic modifiers, histone deacetylases (HDACs). Mammalian cells contain 18 different HDACs, grouped into 4 different classes: 11 HDACs within classes I, II, and IV are Zn^2+^ dependent, while class III is comprised of 7 sirtuins which are NAD^+^ dependent. Class 1 HDACs (HDAC1, HDAC2, HDAC3, and HDAC8) are predominantly localized in the nucleus, have strong deacetylase activity and are expressed in every cell. Class II HDACs shuttle between nucleus and cytoplasm and can be subdivided into class IIa (HDAC4, HDAC5, HDAC7, and HDAC9) and class IIb (HDAC6 and HDAC10). Class IIa enzymes are found in multiprotein complexes and have weak deacetalyse activity, class IIb enzymes are active deacetylases (31).

Acetylation and deacetylation of lysines are post-translational modifications that occur on histone tails, but also on many other non-histone proteins. Choudhary et al. used high-resolution mass spectrometry to identify a total of 1750 acetylated proteins. Lysine acetylation sites overlapped between different cell lines for 60–70% and occurs preferentially in macromolecular complexes affecting the regulation of almost all nuclear, but also many cytoplasmic processes, including chaperone complexes and cytoskeleton remodeling (32).

Acetylation, particularly of histone H3 and histone H4 tails, has almost invariably been linked to a loosened chromatin structure and activation of transcription (33–35). Conversely, histone hypoacetylation correlates with condensed chromatin and transcriptional repression. Histone acetyl transferases (HATs) are enzymes that catalyze the addition of acetyl moieties to histones and other proteins. HAT activity is counter-acted by the activity of HDACs, which remove acetyl groups from lysines in histones and other proteins (36). The function of acetylation on non histone proteins, such as transcription factors, has been elucidated by identification of acetylation of individual proteins (e.g., different STATs, p53, NF-κB) (37–39). The effects of HAT and HDAC activity play direct and indirect roles in a variety of factors including mRNA stability, cell signaling, protein localization, binding, and function and can also prevent or increase proteasomal degradation (40).

## Endogenous Deacetylase Inhibitors

Among SCFAs, butyrate induces the highest acetylation (41). It is generally thought that butyrate inhibits class I and class IIa HDACs but not class IIb (HDAC6 and HDAC10) and sirtuins, however supporting evidence is lacking. One possibility is that butyrate inhibits deacetylation, but not HDAC activity directly (42). However, earlier studies have addressed the potential of SCFAs to alter epigenetic marks in epithelial cells (43). In particular the SCFA butyrate inhibits all class I HDACs. Not only this, it also seems to affect many other epigenetic-related enzymes by regulating the expression of the respective genes encoding HDACs. In bovine epithelial cell cultures, RNA sequencing analyses revealed that the expression of HDACs was modulated by butyrate treatment. Whereas the expression of HCACs 7, 8, and 9 are down-regulated, HDACs 5 and 11 are up-regulated (43). It is to be determined why inhibition of enzymatic activities, seemingly regulates their own expression at the mRNA level. We have summarized earlier data on the effect of SCFA on HDAC activity in different epithelial cell assays in Table 1.

In addition to SCFAs, the microbiome metabolic activity can lead to secretion of metabolites that can interfere with HDAC activity. There are a variety of dietary HDAC inhibitors described earlier, either as dietary component or arising after metabolic activity in the microbiome. Lactate and pyruvate are two of these bacterial components. Lactate itself serves as a substrate for butyrate formation, but has the capacity to inhibit the activity of HDACs, but only in very high concentrations (IC_50_ of 40 mM) (44, 45). These levels of lactate are found in muscle cells only during intense exercise, so it is unlikely that lactate acts as a deacetylase inhibitor in the gut. As such, it is unlikely that the concentrations of SCFAs, besides butyrate and propionate, or bacterial components, like pyruvate and lactate, are high enough to significantly inhibit HDAC function in epithelia, although local fluctuations may render transient HDAC inhibiting properties.

Further examples are isothiocyanates and allyl sulfides, and the foods from which they are derived (46). Sulforaphane is another example, an isothiocyanate, derived from glucoraphanin in broccoli and broccoli sprouts, first identified as a potent inducer of phase 2 detoxification enzymes (46). A common denominator of these compounds is food constituents with chemical structures that contained a spacer “arm” that might fit the HDAC active site, and a functional group that could interact with the buried catalytic zinc atom (47).

## Receptors for SCFAs

Over 90–95% of the produced SCFAs are taken up in the colon and the remaining 5–10% is secreted in the feces. Over 60% of this uptake happens by diffusion of SCFAs across the epithelial membrane, the rest is transported into the cell (16). In human colon, the G-protein coupled nicotinate receptor GPR109A is expressed at the apical membrane of the epithelial layer. Activation of GPR109A by butyrate and other ligands blocked basal and LPS-induced NF-κB activation (48). Butyrate concentration in the lumen is over 10 times higher than what is needed to half-maximal activate this receptor, 1.6 vs. 20 mM, respectively (48).

SCFAs can be transported into the cell by monocarboxylate transporters (MCT), which are coupled to H^+^ transport. Because there is almost no H^+^ gradient over the luminal membrane, this type of transport does not seem to be very active. An alternative transport which is more likely to be the main type of transport is mediated through SLC5A8 (or sodium-coupled MCT, SMCT), which is coupled to Na^+^ over the electrochemical Na^+^ gradient. SLC5A8 is expressed in colonic epithelium at the apical membrane and transports substrates lactate, pyruvate, acetate, propionate, and butyrate (49).

Two additional epithelial receptors for SCFAs were identified. FFAR3 (GPR41) and FFAR2 (GPR43), both G-protein coupled receptors, are activated by high micromolar or millimolar SCFA concentrations, but have different substrate activation potencies (50–52). FFAR2 is expressed in immune cells: mast cells, B lymphocytes, monocytes, eosinophils, and also neutrophils, in which FFAR2 is the only functional receptor for SCFAs in mice. Expression of FFAR2 on immune cells seems to be important in the regulation of immune responses, since mice lacking FFAR2 develop exacerbated inflammation in models for colitis, asthma, and arthritis due to higher production of inflammatory mediators and increased recruitment of immune cells (43, 53). The expression of FFAR2 is also needed for the SCFA-mediated regulation of size and function of the colonic regulatory T-cell pool (54). In addition, FFAR2 functions as a chemotactic receptor, moving neutrophils, and probably other immune cells expressing FFAR2, toward sources of SCFAs (55). FFAR3 mRNA is expressed in many different tissues, but high expression was found primarily in adipose tissue (50, 51). Tazoe et al. identified FFAR3 protein expression in enterocytes and peptide YY containing enteroendocrine cells (56), which also express FFAR2 (57).

**Figure 2:**
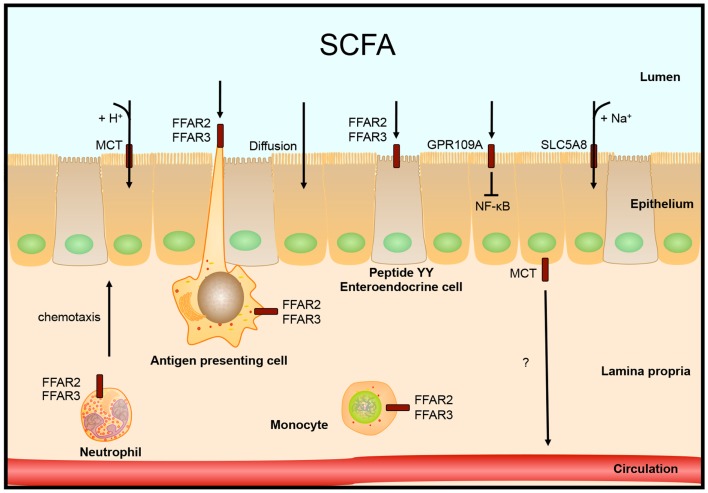
**Intestinal SCFA receptors and transporters**. SCFAs are taken up by the epithelial cells by diffusion, H^+^ coupled transport by monocarboxylate transporters (MCT) or by Na^+^ coupled transport by SLC5A8. Other receptors that are activated by SCFA are localized on colonocytes, peptide YY expressing enteroendocrine cells, or different immune cells. Receptor FFAR2 is involved in neutrophil chemotaxis toward sources of SCFA.

How SCFAs are transported from the colonocytes into the circulation is not entirely clear yet. One possibility is by transport via the MCT. This transporter is expressed at the basolateral membrane, which is in line with this hypothesis (58), see Figure 2.

## HDAC Inhibiting Agents in Inflammatory Bowel Disease

Because of the anti-inflammatory and anti-tumor properties of HDAC inhibition, deacetylase inhibitors have potential for treatment of IBD and IBD associated colorectal cancer, further reviewed in Refs. (59, 60). Increased intake of SCFAs has the capacity to ameliorate colitis disease parameters (53), possibly via the impact on HDAC activity. Glauben et al. tested HDAC inhibitors and SAHA in innate (DSS) and T-cell driven (TNBS) colitis models. In DSS and TNBS models, oral intake of both SAHA and valproic acid reduced several disease parameters. Treatment with VPA in DSS conditions increased acetylation, which was found locally in lamina propria mononuclear cells, but not in liver homogenate or splenocytes (61). A clear impact of HDAC inhibitors is described on the generation of regulatory T cells, an effect that may well contribute to the efficacy of HDAC inhibitors to colitis (62).

## Histone Deacetylases, Colonic Cell Differentiation, and Cancer

Given the ability of HDAC inhibitors to induce growth arrest, maturation, and apoptosis of colon cancer cell lines, it is likely that HDACs themselves play a physiological role in the maintenance of cell proliferation and survival and suppression of IEC maturation. In comparison with HATs, HDACs are of particular interest in medical research because of the ability to inhibit their deacetylase activity. Inhibition of deacetylases leads to hyperacetylation and results in a block of proliferation in tumor cells, by a variety of mechanisms, including the induction of differentiation, apoptosis, and transcriptional upregulation of tumor suppressors (63). Two chemical HDAC inhibitors, vorinostat and romidepsin, have been approved by the FDA for treatment of cutaneous T-cell lymphoma (46). HDAC inhibitors also negatively effect tumorigenesis by affecting angiogenesis and modulating immune responses (64). In addition, SAHA has prominent effects on the inflammatory response (65). This gives rise to potential use in a range of other diseases, including chronic inflammatory disease like IBD (66).

The modulating effects of HDAC inhibition on epithelial cell differentiation can have major effects on the development of colon cancers. Functional genetic screenings to elucidate potential pathways targeted by HDAC inhibitors were described earlier (67). In these screens overexpression of genes involved in RA signaling selectively rendered tumor cells resistant to treatment with HDAC inhibitors. In these resistant cells, overexpression of two genes allowed cells to bypass proliferation arrest and apoptosis imposed by HDAC inhibitors: RA receptor alpha (RARα) and preferentially expressed antigen of melanoma (67). The latter gene encodes a tumor antigen and has been identified as a repressor of RA signaling (68). Hence, HDAC inhibitor treatment caused de-repression of RA target genes in these cells, suggesting a role for RA signaling in the anti-cancer effects induced by HDAC inhibitors.

This is particularly interesting in the light of effects of butyrate on epithelial modulation of cell proliferation, survival, and apoptosis (16, 43). In particular HDAC3 was put forward because overexpression of HDAC overcomes the butyrate repression of p21, a potent cyclin-dependent kinase inhibitor that functions as a regulator of cell cycle progression at G1 (69, 70). Additionally, protein expression of HDAC3 was significantly up-regulated in a panel of human colon tumors compared with adjacent normal mucosa and in small intestinal adenomas derived from APC mutant mice epithelia, establishing a link between HDAC3 expression and intestinal cell transformation. From these findings it is expected that aberrant expression of HDAC3 and other class I HDACs play a role in the progression of colon cancer. HDAC3 activity may be dependent on the activity of class IIa HDAC4 protein, as Sp1-dependent targeting of HDAC4 to the proximal p21 promoter in colon cancer cells was shown (70). Hence, repression of the activity of the proximal p21 promoter is likely mediated by HDAC4 through association with the catalytically active HDAC3, within the N-CoR/SMRT co-repressor complex (69, 71). Furthermore, it has been shown that HDAC4 acts as a “scaffold” protein with the HDAC3–NCo-R/SMRT complex without contributing to the overall deacetylase activity of the complex, consistent with the weak catalytic activity of HDAC4 and other class IIa HDACs (71, 72).

## Future Perspectives

The human intestinal flora has an important role in homeostasis in health and disease, not only because the help to digest food, but also because of the secretion of many metabolites. Differences in diversity were found between feces from healthy individuals and feces from patients with various disease states, obesity, type 2 diabetes, IBD, allergies (11). It remains unknown whether the shifts in flora are consequential of disease or whether they also have a causative nature. Interestingly, in feces from IBD patients, the diversity of butyrate-producing bacteria is lower than in feces of healthy controls (13, 14). Even though fecal samples do not reflect the actual intestinal flora, such a shift has the potential to affect the intestine considerably by the altering metabolite and SCFA secretion (73).

In line with this, it will also be interesting to see to what extend the SCFA levels and HDAC-mediated processes can be modulated by administration of prebiotics and probiotics (74–76). In patients with chronic pouchitis and ulcerative colitis patients in remission, probiotic treatment was effective in the prevention of relapse. However, additional controlled clinical trials are needed to fully appreciate the potential of prebiotic or probiotic administration in IBD (77–79).

Other promising studies that are ongoing are fecal microbiota transplantation studies performed in different diseased settings, including obesity, metabolic syndrome, IBD, and in recurrent *Clostridium difficile* infections (80). In a recent trail with patients with recurrent *C. difficile* infections, 81.3% of the patients cured from infection after a single infusion of donor feces, which was substantially higher than the 30% with standard antibiotic treatment. Microbiota diversity of the patients increased 2 weeks after infusion to reach levels of donors (81). In another study, intestinal microbiota transfer was used to increase insulin sensitivity in patients with metabolic syndrome. Again, microbial diversity was increased after infusion, among them were different strains of butyrate-producing bacteria (82). Careful analysis of these therapies can possible give an indication whether the altered microbiota composition is the consequence of chronic ongoing inflammation or whether it has a more causative nature.

## Conflict of Interest Statement

W. J. de Jonge receives funding from Mead Johnson Nutrition and GlaxoSmithKline Research.
