# Erratum: Dietary Inhibitors of Histone Deacetylases in Intestinal Immunity and Homeostasis

**DOI:** 10.3389/fimmu.2013.00414

**Published:** 2013-11-27

**Authors:** R. Schilderink, C. Verseijden, W. J. de Jonge

**Affiliations:** ^1^Tytgat Institute for Liver and Intestinal Research, Department of Gastroenterology and Hepatology, Academic Medical Center, Amsterdam, Netherlands

Figure 1 of Dietary inhibitors of histone deacetylases in intestinal immunity and homeostasis has incorrect labels. Attached is an updated and correct version of this figure.

**Figure 1:**
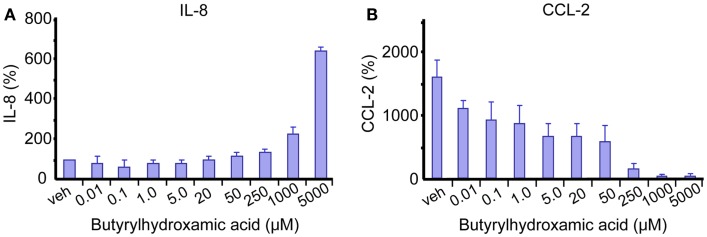
**The effects of SCFA-derivative butyrylhydroxamic acid on epithelial cell responses in Caco-2 enterocytes**. Cells were left either unstimulated **(A)** or stimulated with IL1β (10 ng/mL for 24 h); **(B)**, in the presence of indicated butyrylhydroxamic acid concentrations. Secreted levels of IL-8 and CCL-2 were measured and plotted as percentages to vehicle treated unstimulated cells [veh graph **(A)**].

